# A Generalized Simplest Equation Method and Its Application to the Boussinesq-Burgers Equation

**DOI:** 10.1371/journal.pone.0126635

**Published:** 2015-05-14

**Authors:** Bilige Sudao, Xiaomin Wang

**Affiliations:** College of Sciences, Inner Mongolia University of Technology, Hohhot, Inner Mongolia, PR China; Centro de Investigacion Cientifica y Educacion Superior de Ensenada, MEXICO

## Abstract

In this paper, a generalized simplest equation method is proposed to seek exact solutions of nonlinear evolution equations (NLEEs). In the method, we chose a solution expression with a variable coefficient and a variable coefficient ordinary differential auxiliary equation. This method can yield a B*ä*cklund transformation between NLEEs and a related constraint equation. By dealing with the constraint equation, we can derive infinite number of exact solutions for NLEEs. These solutions include the traveling wave solutions, non-traveling wave solutions, multi-soliton solutions, rational solutions, and other types of solutions. As applications, we obtained wide classes of exact solutions for the Boussinesq-Burgers equation by using the generalized simplest equation method.

## 1 Introduction

It is important to seek more exact solutions of nonlinear evolution equations (NLEEs) in mathematical physics. In past decades, many powerful methods have been presented such as the inverse scattering method [1], the Darboux transformation [2], the Hirota bilinear method [3], the Painlevé expansion method [4], the Bäcklund transformation method [5, 6], the multilinear variable separation method [7], the homogeneous balance method [8], the Jacobi elliptic function expansion method [9], the tanh-function method [10, 11], the F-expansion method [12], the auxiliary equation method [13], the sub-ODE method [14], the Exp-function method [15], the (*G*′/*G*)-expansion method [16, 17], the simplest equation method [18, 19]. Thousands of examples have shown that these methods are powerful for obtaining exact solutions of NLEEs, especially, for traveling wave solutions. Up to now, a unified method that can be used to deal with all types of NLEEs has not been discovered. Hence, developing new method and finding more general exact solutions of NLEEs have drawn a lot of interests of a diverse group of scientists.

Particularly, N.A. Kudryashov first proposed the simplest equation method and showed that it is powerful for finding analytic solutions of NLEEs [18, 19]. Two basic ideas are at the heart of the approach. The first idea is to apply the simplest nonlinear differential equations (the Riccati equation, the equation for the Jacobi elliptic faction, the equation for the Weierstrass ellipic function and so on) that have lesser order than the equation studied. The second idea is to use possible singularities of general solution for the equation studied. Recently, there are many applications and generalizations of the method [20–25].

In the present paper, based on a solution expression with a variable coefficient and variable coefficient auxiliary equation (a special simplest nonlinear differential equation), we proposed a generalized simplest equation method to seek exact solutions of NLEEs. This method can yield infinite number of exact solutions of NLEEs. For illustration, we apply this method to the Boussinesq-Burgers equation and successfully find many different types of exact solutions. These solutions include traveling wave solutions, non-traveling wave solutions, multi-soliton solutions, rational solutions and other types of solutions.

The rest of the paper is organized as follows. In Section 2, we describe the generalized simplest equation method to look for exact solutions of NLEEs. In Section 3, we firstly construct the most general form exact solutions and the exact traveling wave solutions of the Boussinesq-Burgers equation by using our method. Secondly, we obtain the Bäcklund transformation between the Boussinesq-Burgers equation and the constraint equation. Thirdly, we give some concrete new exact solutions of the Boussinesq-Burgers equation. In Section 4, we give some discussions and conclusion remarks.

## 2 Description of the generalized simplest equation method

We consider a nonlinear evolution equation (NLEE)

$$\begin{array}{c} P(u,u_{t},u_{x},u_{tx},u_{tt},u_{xx},\cdots )=0, \end{array}$$ (1)

where *u* = *u*(*t*, *x*) is an unknown function, *t*, *x* are two independent variables, *P* is a polynomial in *u* and its various partial derivatives.

We consider a generalized simplest equation method for obtaining exact solutions to the given NLEE Eq (1), which is described in the following five steps.

**Step 1**. Suppose that the solutions for Eq (1) can be expressed in the following general form

$$\begin{array}{c} u\left(t,x\right)=\sum_{i=0}^{M}\alpha_{i}\left(t,x\right)\phi^{i}\left(\xi \right), \end{array}$$ (2)

where *α*_*i*_(*t*, *x*)(*i* = 0, 1, ⋯, *M*) and *ξ* = *ξ*(*t*, *x*) are all functions of *t*, *x* to be determined later, *ϕ* = *ϕ*(*ξ*) satisfies our auxiliary equation (a first order ordinary differential equation (ODE) with variable coefficients):

$$\begin{array}{c} \xi^{2}\left(\phi^{\prime }+\phi^{2}\right)+\delta \xi \phi +\nu =0. \end{array}$$ (3)

It has three types of general solution as follows

$$\begin{array}{ccc} & & \phi_{1}\left(\xi \right)=\varepsilon \cdot \frac{[\left(1-\delta \right)c_{1}-2\eta c_{2}]\sin\left(\eta \ln|\xi |\right)+[2\eta c_{1}+\left(1-\delta \right)c_{2}]\cos\left(\eta \ln|\xi |\right)}{2\xi [c_{1}\sin(\eta \ln|\xi |+c_{2}\cos\left(\eta \ln|\xi |\right)]};\;\mathrm{if}\;s>1 \end{array}$$ (4)

$$\begin{array}{ccc} & & \phi_{2}\left(\xi \right)=\varepsilon \cdot \frac{c_{1}\left(2\eta -\delta +1\right){\left|\xi \right|}^{2\eta }-c_{2}\left(2\eta +\delta -1\right)}{2|\xi |(c_{1}{\left|\xi \right|}^{2\eta }+c_{2})};\;\mathrm{if}\;s<1 \end{array}$$ (5)

$$\begin{array}{ccc} & & \phi_{3}\left(\xi \right)=\varepsilon \cdot \frac{\left(1-\delta \right)(c_{1}+c_{2}\ln|\xi |)+2c_{2}}{2\xi (c_{1}+c_{2}\ln\left|\xi \right|)},\;\mathrm{if}\;s=1 \end{array}$$ (6)

and

$$\begin{array}{c} \varepsilon =\{\begin{array}{c} \;\;1,\;\;\;\;\;\text{if}\;\;\xi >0,\\ -1,\;\;\;\;\;\text{if}\;\;\xi <0, \end{array} \end{array}$$ (7)

where *c*_1_, *c*_2_, *δ*, *ν* are constants and

$$s=2\delta -\delta^{2}+4\nu ,\;\eta =\frac{1}{2}{\left|1-s\right|}^{1/2}.$$

**Step 2**. The positive number *M* can be determined by considering the homogeneous balance between the highest order derivatives and nonlinear terms appearing in Eq (1).

**Step 3**. By substituting Eq (2) into Eq (1) and using Eq (3), collecting all terms with the same order of *ϕ* together, the left-hand side of Eq (1) is converted into another polynomial in *ϕ*. Equating each coefficient of powers of *ϕ* to zero, yields a set of over-determined partial differential equations for *α*_*i*_(*t*, *x*)(*i* = 0, 1, ⋯, *M*) and *ξ*(*t*, *x*).

**Step 4**. By solving the system of over-determined partial differential equations in Step 3, we obtain *α*_*i*_(*t*, *x*)(*i* = 0, 1, ⋯, *M*) and *ξ*(*t*, *x*).

**Step 5**. By substituting *α*_*i*_(*t*, *x*)(*i* = 0, 1, ⋯, *M*), *ξ*(*t*, *x*) and the general solutions Eqs (4), (5) and (6) of Eq (3) into Eq (2), we can obtain more exact solutions of the Eq (1).

**Remark 1**: The solution expression Eq (2) with functions *α*_*i*_(*t*, *x*)(*i* = 0, 1, ⋯, *M*) and *ξ*(*t*, *x*) can yield more general form exact solutions rather than just traveling wave solutions.

**Remark 2**: It is the first time that Eq (3) is used as an auxiliary equation for obtaining exact solutions of NLEEs.

**Remark 3**: It is emphasized that in our designs Eqs (2) and (3), the parameters *δ*, *ν* will play the role of adjusters in solving the constraint equation and in obtaining a transformation between Eq (1) and linear heat equation (see Section 3).

## 3 Application of the Method

### The exact solutions to the Boussinesq-Burgers equation

In this section, we are aimed to first give the most general form exact solutions, then we will determine the exact traveling wave and non-traveling wave solutions for the Boussinesq-Burgers equation [26–32]

$$\begin{array}{c} u_{t}+2uu_{x}-\frac{1}{2}v_{x}=0, \end{array}$$ (8)

$$\begin{array}{c} v_{t}+2{\left(uv\right)}_{x}-\frac{1}{2}u_{xxx}=0. \end{array}$$ (9)

#### The general form exact solutions

In the subsection, we solve the most general form exact solutions of Eqs (8) and (9).

**Step 1**. The solution expressions of Eqs (8) and (9) are taken as Eq (2).

**Step 2**. Considering the homogeneous balance between the highest order derivatives *v*_*x*_(*u*_*xxx*_) and nonlinear terms *uu*_*x*_(*uv*_*x*_) in Eqs (8) and (9), we get balance numbers *M* = 1 for *u* and *N* = 2 for *v*. Thus, we can denote the solutions of Eqs (8) and (9) in the form

$$\begin{array}{c} u\left(t,x\right)=a_{0}\left(t,x\right)+a_{1}\left(t,x\right)\phi \left(\xi \right), \end{array}$$ (10)

$$\begin{array}{c} v\left(t,x\right)=b_{0}\left(t,x\right)+b_{1}\left(t,x\right)\phi \left(\xi \right)+b_{2}\left(t,x\right)\phi^{2}\left(\xi \right), \end{array}$$ (11)

where functions *a*_0_(*t*, *x*), *a*_1_(*t*, *x*), *b*_0_(*t*, *x*), *b*_1_(*t*, *x*), *b*_2_(*t*, *x*) and *ξ* = *ξ*(*t*, *x*) are to be determined later and *ϕ* = *ϕ*(*ξ*) satisfies Eq (3).

**Step 3**. We substitute Eqs (10) and (11) along with the auxiliary Eq (3) into Eqs (8) and (9) and collect all terms with the same order of *ϕ*. As a result, the left-hand sides of Eqs (8) and (9) are converted into another polynomials in *ϕ*. Equating each coefficient of powers of *ϕ* to zero, we obtain a set of over-determined partial differential equations for *a*_0_(*t*, *x*), *a*_1_(*t*, *x*), *b*_0_(*t*, *x*), *b*_1_(*t*, *x*), *b*_2_(*t*, *x*) and *ξ*(*t*, *x*).

**Remark 4**: Unlike *pure* algebraic equations used in the standard auxiliary method, here we obtain differential equations for *a*_*i*_(*t*, *x*), *b*_*j*_(*t*, *x*) and *ξ*(*t*, *x*). This provides us with more general types of exact solutions to Eqs (8) and (9).

**Step 4**. Solving the differential system obtained in Step 3 by Mathematica, we obtain *a*_0_(*t*, *x*), *a*_1_(*t*, *x*), *b*_0_(*t*, *x*), *b*_1_(*t*, *x*), *b*_2_(*t*, *x*) expressed by *ξ*(*t*, *x*) and *δ*, *ν* as follows:

$$\begin{array}{ccc} a_{0}\left(t,x\right) & = & \frac{\xi \left(\mp \xi_{xx}-2\xi_{t}\right)\pm \delta \xi_{x}^{2}}{4\xi \xi_{x}},\;a_{1}\left(t,x\right)=\pm \frac{1}{2}\xi_{x},\\ b_{0}\left(t,x\right) & = & \frac{\left(\delta +2\nu \right)\xi_{x}^{4}-\delta \xi \xi_{x}^{2}\xi_{xx}-\xi^{2}\xi_{xx}\left(\xi_{xx}\pm 2\xi_{t}\right)+\xi^{2}\xi_{x}\left(\xi_{xxx}\pm 2\xi_{xt}\right)}{4\xi^{2}\xi_{x}^{2}},\\ b_{1}\left(t,x\right) & = & \frac{\delta \xi_{x}^{2}-\xi \xi_{xx}}{2\xi },\;b_{2}\left(t,x\right)=\frac{1}{2}\xi_{x}^{2}, \end{array}$$ (12)

where *ξ* = *ξ*(*t*, *x*) satisfies the equation

$$\begin{array}{ccc} & & s(\xi \xi_{xx}-\xi_{x}^{2})\xi_{x}^{4}+\xi^{3}[(\xi_{xxxx}\pm 4\xi_{txx}+4\xi_{tt})\xi_{x}^{2}+4(\mp \xi_{xx}-\xi_{t})(2\xi_{tx}\pm \xi_{xxx})\xi_{x}\\ & & \;+(4\xi_{t}^{2}+3\xi_{xx}^{2}\pm 8\xi_{t}\xi_{xx})\xi_{xx}]=0. \end{array}$$ (13)

Eq (13) is called **the constraint equation** of *ξ*.

Here for obtaining more general types of solutions, we consider the case of *ξ*_*x*_ ≠ 0. Any solution of the constraint Eq (13) leads to a group of coefficients Eq (12) which together with Eqs (4)–(6) result in three classes of exact solutions Eqs (10) and (11) of Eqs (8) and (9) as follows:

$$\begin{array}{c} \left\{ \begin{array}{c} u_{i}\left(t,x\right)=a_{0}\left(t,x\right)+a_{1}\left(t,x\right)\phi_{i}\left(\xi \right),\\ v_{i}\left(t,x\right)=b_{0}\left(t,x\right)+b_{1}\left(t,x\right)\phi_{i}\left(\xi \right)+b_{2}\left(t,x\right)\phi_{i}^{2}\left(\xi \right), \end{array}\right. \end{array}$$ (14)

in which *i* = 1 for *s* > 1, *i* = 2 for *s* < 1 and *i* = 3 for *s* = 1.

Therefore, the solution expressions Eq (14) have established a Bäcklund transformation between Eqs (8) and (9) and constraint Eq (13). Because of the Bäcklund transformation, the method can give infinite number of exact solutions for the considering NLEEs immediately.

Seen from the formulae Eq (14), if *ξ*(*t*, *x*) is a solution of Eq (13) with the form *ξ* = *ξ*(*x*−*Vt*), then it yields exact traveling wave solutions of Eqs (8) and (9). While if *ξ* ≠ *ξ*(*x*−*Vt*), then it yields exact non-traveling wave solutions of Eqs (8) and (9). Hence the formulae Eq (14) provide us with abundance of general form exact solutions to Eqs (8) and (9) once the solutions of Eq (13) are given.

**Step 5**. In the following, we determine the exact traveling wave and non-traveling wave solutions of Eqs (8) and (9) through exactly solving Eq (13).

#### The exact traveling wave solutions

In this subsection, we find the exact traveling wave solutions of Eqs (8) and (9) through obtaining specific solutions of Eq (13) formed by *ξ*(*t*, *x*) = *ξ*(*x*−*Vt*). In this case, Eq (13) becomes

$$\begin{array}{ccc} & & s\left[\xi^{\prime }{\left(z\right)}^{2}-\xi \left(z\right)\xi^{\prime \prime }\left(z\right)\right]\xi^{\prime }{\left(z\right)}^{4}+\left[4\xi^{\prime }\left(z\right)\xi^{\prime \prime }\left(z\right)\xi^{\left(3\right)}\left(z\right)-\xi^{\prime }{\left(z\right)}^{2}\xi^{\left(4\right)}\left(z\right)-3\xi^{\prime \prime }{\left(z\right)}^{3}\right]\xi {\left(z\right)}^{3}=0, \end{array}$$ (15)

where *z* = *x*−*Vt*.

With the transformation

$$\begin{array}{c} \xi \left(t,x\right)=\xi \left(z\right),\xi^{\prime }\left(z\right)=Y\left(\xi \right), \end{array}$$ (16)

Eq (15) becomes the third order linear ODE with variable coefficients

$$\begin{array}{c} \xi^{3}Y^{\left(3\right)}\left(\xi \right)+s[\xi Y^{\prime }\left(\xi \right)-Y\left(\xi \right)]=0, \end{array}$$

which has general solutions as follows:

$$\begin{array}{c} Y\left(\xi \right)=\{\begin{array}{c} \left|\xi \right|[d_{1}+d_{2}\cos(\delta_{0}\ln\left|\xi \right|)+d_{3}\sin(\delta_{0}\ln\left|\xi \right|)],\;\mathrm{if}\;\;\delta_{0}^{2}=s-1>0,\\ \left|\xi \right|(d_{1}{\left|\xi \right|}^{-\delta_{0}}+d_{2}{\left|\xi \right|}^{\delta_{0}}+d_{3}),\;\mathrm{if}\;\;\delta_{0}^{2}=1-s>0,\\ \left|\xi \right|(d_{1}+d_{2}\ln|\xi |+d_{3}\ln^{2}\left|\xi \right|),\;\;\mathrm{if}\;\;\delta_{0}^{2}=1-s=0, \end{array} \end{array}$$ (17)

where *d*_1_, *d*_2_ and *d*_3_ are arbitrary constants. For a nontrivial solution, these constants should not be equal to zero simultaneously. Correspondingly, from Eqs (16) and (3), we have twelve kinds of exact solutions for Eq (13) respectively, which are summarized in the following three cases and their subcases.

**Case 1**: *s* > 1. In this case, there are three kinds of exact solutions of Eq (13) shown in the following subcases.

**Subcase 1.1**: $d_{2}^{2}+d_{3}^{2}-d_{1}^{2}>0$, $A=\sqrt{d_{2}^{2}+d_{3}^{2}-d_{1}^{2}},A_{1}=d_{2}+\exp[d_{3}(z+d_{4})\delta_{0}]$,
  $$\begin{array}{c} \xi \left(z\right)=\{\begin{array}{c} \varepsilon \;\exp[-\frac{2\varepsilon }{\delta_{0}}\arctan(\frac{A\tanh(\frac{A}{2}\delta_{0}\left(z+d_{4}\varepsilon \right))+d_{3}\varepsilon }{d_{1}-d_{2}})],\;\text{if}\;d_{1}\neq d_{2}\\ \varepsilon \;\exp[\frac{2}{\delta_{0}}\arccos(\frac{\pm d_{3}}{\sqrt{d_{2}^{2}+d_{3}^{2}+\exp\left(d_{3}\delta_{0}\left(z+d_{4}\right)\right)\left(d_{2}+A_{1}\right)}})],\;\text{if}\;d_{1}=d_{2} \end{array} \end{array}$$ (18)
**Subcase 1.2**: $d_{2}^{2}+d_{3}^{2}-d_{1}^{2}<0$,$A^{2}=d_{1}^{2}-d_{2}^{2}-d_{3}^{2},d_{1}\neq d_{2}$,
  $$\begin{array}{c} \xi \left(z\right)=\varepsilon \exp[\frac{2}{\delta_{0}}\arctan(\frac{A\tan(-\frac{A}{2}\delta_{0}\varepsilon \left(z+d_{4}\right))+d_{3}}{d_{2}-d_{1}})]. \end{array}$$ (19)
**Subcase 1.3**: $d_{2}^{2}+d_{3}^{2}-d_{1}^{2}=0$,
  $$\begin{array}{c} \xi \left(z\right)=\exp[-\varepsilon \;\arccos(\frac{\pm K_{2}\left(z\right)\left(d_{1}+\varepsilon d_{2}K_{1}\left(z\right)\right)+d_{1}K_{1}^{2}\left(z\right)\left(d_{1}+d_{1}K_{1}^{2}\left(z\right)-2\varepsilon d_{2}K_{1}\left(z\right)\right)d_{3}^{3}}{d_{3}K_{1}\left(z\right)\left(d_{1}^{4}\left(1+K_{1}^{4}\left(z\right)\right)+2\left(d_{3}^{4}-d_{2}^{4}\right)K_{1}^{2}\left(z\right)\right)})], \end{array}$$ (20)
  where
  $$\begin{array}{ccc} K_{1}\left(z\right) & = & d_{3}\delta_{0}\left(z+d_{4}\right),\\ K_{2}\left(z\right) & = & d_{1}^{2}d_{3}^{2}K_{1}^{2}\left(z\right)[d_{1}^{4}K_{1}^{6}\left(z\right)+2\theta \varepsilon {|d_{1}|}^{3}d_{2}K_{1}^{5}\left(z\right)+d_{1}^{2}d_{2}^{2}+4\varepsilon d_{1}d_{2}^{3}K_{1}^{3}\left(z\right)+2\varepsilon d_{1}d_{2}K_{1}\left(z\right)\left(d_{3}^{2}-d_{2}^{2}\right)\\ & & +\;K_{1}^{2}\left(z\right)\left(-d_{2}^{4}-4d_{2}^{2}d_{3}^{2}+d_{3}^{4}\right)+K_{1}^{4}\left(z\right)\left(-d_{2}^{4}+d_{2}^{2}d_{3}^{2}+2d_{3}^{4}\right)]. \end{array}$$
  and
  $$\begin{array}{c} \theta =\{\begin{array}{c} -1,\;\;\;\;\;\text{if}\;\;d_{1}>0,\\ \;\;1,\;\;\;\;\;\text{if}\;\;d_{1}<0. \end{array} \end{array}$$

**Case 2**: *s* < 1. In this case, there are five kinds of exact solutions of Eq (13) shown in the following subcases.

**Subcase 2.1**: $4d_{1}d_{2}-d_{3}^{2}>0,A=\sqrt{4d_{1}d_{2}-d_{3}^{2}},$
  $$\begin{array}{c} \xi \left(z\right)=\varepsilon {\left[\frac{-2d_{1}}{\varepsilon A\tan(\frac{\delta_{0}}{2}\left(z+d_{4}\right)A)+d_{3}}\right]}^{\frac{1}{\delta_{0}}}. \end{array}$$ (21)
**Subcase 2.2**: $4d_{1}d_{2}-d_{3}^{2}<0,d_{2}\neq 0,A^{2}=d_{3}^{2}-4d_{1}d_{2},$
  $$\begin{array}{c} \xi \left(z\right)=\varepsilon {\left[-\frac{\varepsilon A\tanh(\frac{\delta_{0}}{2}\left(z+d_{4}\right)A)+d_{3}}{2d_{2}}\right]}^{\frac{1}{\delta_{0}}}. \end{array}$$ (22)
**Subcase 2.3**: $4d_{1}d_{2}-d_{3}^{2}<0,d_{2}=0,d_{3}\neq 0,$
  $$\begin{array}{c} \xi \left(z\right)=\varepsilon {\left[\frac{\exp\left(\varepsilon d_{3}\delta_{0}\left(z+d_{4}\right)\right)-d_{1}}{d_{3}}\right]}^{\frac{1}{\delta_{0}}}. \end{array}$$ (23)
**Subcase 2.4**: $4d_{1}d_{2}-d_{3}^{2}=0,d_{1}d_{2}\neq 0,d_{3}\neq 0,$
  $$\begin{array}{c} \xi \left(z\right)=\varepsilon {\left[-\frac{2d_{1}}{d_{3}}-\frac{4\varepsilon d_{1}}{d_{3}^{2}\left(z+d_{4}\right)\delta_{0}}\right]}^{\frac{1}{\delta_{0}}}. \end{array}$$ (24)
**Subcase 2.5**: $4d_{1}d_{2}-d_{3}^{2}=0,d_{1}=0,d_{2}\neq 0,$ i.e., *d*_1_ = *d*_3_ = 0,
  $$\begin{array}{c} \xi \left(z\right)=\varepsilon {\left[-\frac{\varepsilon }{d_{2}\delta_{0}\left(z+d_{4}\right)}\right]}^{\frac{1}{\delta_{0}}}. \end{array}$$ (25)

**Case 3**: *s* = 1. In this case, there are four kinds of solutions of Eq (13) shown in the following subcases.

**Subcase 3.1**: $4d_{1}d_{3}-d_{2}^{2}>0,d_{3}\neq 0,$
  $$\begin{array}{c} \xi \left(z\right)=\varepsilon \exp[\frac{\varepsilon \sqrt{4d_{1}d_{3}-d_{2}^{2}}\tan\left(\frac{1}{2}\sqrt{4d_{1}d_{3}-d_{2}^{2}}\left(z+d_{4}\right)\right)-d_{2}}{2d_{3}}]. \end{array}$$ (26)
**Subcase 3.2**: $4d_{1}d_{3}-d_{2}^{2}<0,d_{3}\neq 0,A^{2}=d_{2}^{2}-4d_{1}d_{3},$
  $$\begin{array}{c} \xi \left(z\right)=\varepsilon \exp[-\frac{\varepsilon A\tanh\left(\frac{1}{2}A\left(z+d_{4}\right)\right)+d_{2}}{2d_{3}}]. \end{array}$$ (27)
**Subcase 3.3**: $4d_{1}d_{3}-d_{2}^{2}<0,d_{3}=0,d_{2}\neq 0,$
  $$\begin{array}{c} \xi \left(z\right)=\{\begin{array}{c} \exp[\frac{\exp\left(d_{2}\left(z+d_{4}\right)\right)-d_{1}}{d_{2}}],\;\text{if}\;\xi \left(z\right)>0,\\ -\exp[\frac{i\exp\left(-d_{2}\left(z+d_{4}\right)\right)-d_{1}}{d_{2}}],\;\text{if}\;\xi \left(z\right)<0,\;i^{2}=-1. \end{array} \end{array}$$ (28)
**Subcase 3.4**: $4d_{1}d_{3}-d_{2}^{2}=0,d_{3}=0,$ i.e., *d*_2_ = *d*_3_ = 0,
  $$\begin{array}{c} \xi \left(z\right)=d_{4}\exp(\varepsilon d_{1}z). \end{array}$$ (29)

In all of above cases, *d*_4_ is an additional arbitrary constant of integration and *ɛ* is given in Eq (7).

Substituting the obtained solutions *ξ* = *ξ*(*x*−*Vt*) in all of above cases Eqs (18)–(29) into Eqs (4)–(6) and (12) and assembling them in Eq (14), we obtain twelve kinds of exact traveling wave solutions of Eqs (8) and (9). Here we omit the duplicate and long expressions of these solutions.

**Remark 5**: A feature of these solutions is that they are multi-composite solutions, i.e., they are constructed by compounding several elementary functions. For example, in the case *s* > 1, the solutions Eqs (18)–(20) are the compounding of five elementary functions exp, arctan, tanh, tan, arccos etc. These solutions can not be obtained by tanh-function method, the (*G*′/*G*)-expansion method and other method given [6] etc. The solution Eqs (21), (22), (26) and (27) have included soliton solutions when the parameters are taken properly. The solutions Eqs (24) and (25) are rational or irrational solutions at some values of these parameters appeared in these solutions. These already significantly expand the set of exact solutions for Eqs (8) and (9).

### A transformation between Eqs (8) and (9) and a linear heat equation

It is observed that if the *t*-derivative is equal to second order *x*-derivative, then each monomial term in Eq (13) has the same sum of *x*-derivative orders. This hints a relationship between Eq (13) and a kind of heat equation. In fact, Eq (13) can be written as

$$\begin{array}{c} s(\xi \xi_{xx}-\xi_{x}^{2})\xi_{x}^{4}+\xi^{3}\left[4P(P\pm 2\xi_{xx})\xi_{xx}-4P_{x}(\xi_{xx}\pm 2P)\xi_{x}+2(2P_{t}-2aP_{x}\pm P_{xx})\xi_{x}^{2}\right]=0, \end{array}$$ (30)

where *P* = *ξ*_*t*_−*τξ*_*xx*_−*aξ*_*x*_ for arbitrary constant *a*, and

$$\begin{array}{c} \tau =\{\begin{array}{c} -1/2,\;\;\;\;\;\text{if}\;\;a_{1}\left(t,x\right)=\xi_{x}/2,\\ \;\;1/2,\;\;\;\;\;\text{if}\;\;a_{1}\left(t,x\right)=-\xi_{x}/2. \end{array} \end{array}$$ (31)

In order to get most general form exact solutions, we consider the following two cases.

**Case I**. For $\xi \xi_{xx}-\xi_{x}^{2}=0$ and *s* = 2*δ*−*δ* ^2^+4*ν*.

In this case, by solving Eq (13) we have *ξ*(*t*, *x*) = *d*_4_exp[(*d*_2_ *x*+*d*_3_)/(*t*+*d*_1_)], which results in exact solutions of Eqs (8) and (9) given by Eq (14) with Eq (12) as follows

$$\begin{array}{ccc} u_{i}\left(t,x\right) & = & \frac{2d_{3}+d_{2}\left[2x\pm d_{2}\left(\delta -1\right)\right]}{4d_{2}\left(d_{1}+t\right)}\pm \frac{d_{2}}{2(d_{1}+t)}\cdot \xi \left(t,x\right)\phi_{i}\left(\xi \right),\\ v_{i}\left(t,x\right) & = & \mp \frac{d_{1}+t-d_{2}^{2}\nu }{2{\left(d_{1}+t\right)}^{2}}+\frac{d_{2}^{2}\left(\delta -1\right)}{2{\left(d_{1}+t\right)}^{2}}\cdot \xi \left(t,x\right)\phi_{i}\left(\xi \right)+\frac{d_{2}^{2}}{2{\left(d_{1}+t\right)}^{2}}\cdot \xi^{2}\left(t,x\right)\phi_{i}^{2}\left(\xi \right), \end{array}$$ (32)

in which *i* = 1 for *s* > 1, *i* = 2 for *s* < 1 and *i* = 3 for *s* = 1. *ϕ*_1_(*ξ*), *ϕ*_2_(*ξ*) and *ϕ*_3_(*ξ*) are given in Eqs (4)–(6). *d*_1_, *d*_2_, *d*_3_, *d*_4_ are arbitrary constants.

**Case II**. For *s* = 2*δ*−*δ* ^2^+4*ν* = 0.

In this case, Eq (13) or Eq (30) admits the solutions *ξ*(*t*, *x*) of the linear heat equation

$$\begin{array}{c} \xi_{t}=\tau \xi_{xx}+a\xi_{x}, \end{array}$$ (33)

with a conductive term *aξ*_*x*_ for arbitrary constant *a*.

It is interesting that a transformation in the form of Eq (14) between Eqs (8) and (9) and the linear heat Eq (35) has been found. Using the transformation, we will obtain infinite number of exact solutions of Eqs (8) and (9). That means each solution of the heat Eq (35) (such as those given in the following Eq (34)) yields a set of exact solutions of Eqs (8) and (9). Moreover, the connection provides us with an insight into integrability of Eqs (8) and (9). For example, using the transformation, we can investigate the multi-soliton solutions, rational solutions and other types of solutions to the Boussinesq-Burgers equation.

As we know, the heat Eq (35) has infinite number of specific known exact solutions. Some representatives of them are listed as follows:

$$\begin{array}{ccc} \xi (t,x) & = & \frac{A}{\sqrt{t}}\exp\left(-\frac{{\left(x+at\right)}^{2}}{4\tau t}\right)+B,\;\;\left(t,x\right)\in R^{2},\\ \xi (t,x) & = & A\exp\left(-\tau k^{2}t\right)\cos\left(k\left(x+at\right)+B\right)+C,\;\;\left(t,x\right)\in R^{2},\\ \xi (t,x) & = & A\exp\left(-k\left(x+at\right)\right)\cos\left(kx+k\left(a-2\tau k\right)t+B\right)+C,\;\;\left(t,x\right)\in R^{2},\\ \xi (t,x) & = & c_{0}\mathrm{erfc}\left(\frac{x+at}{2\sqrt{\tau t}}\right),\;\;t>0,x+at>0,\tau >0,\\ \xi (t,x) & = & \sum_{n=1}^{+\infty }c_{n}\sin\left(\frac{n\pi (x+at)}{L}\right)\exp\left(-\tau {\left(\frac{n\pi }{L}\right)}^{2}t\right),\;\;t>0,0\leq x+at\leq L,L>0,\\ \xi (t,x) & = & \frac{1}{2\sqrt{\pi \tau t}}\int_{-\infty }^{+\infty }\omega \left(\theta \right)\exp\left(-\frac{{\left(x+at-\theta \right)}^{2}}{4\tau t}\right)d\theta ,\;\;t>0,x\in R,\tau >0, \end{array}$$ (34)

where *A*, *B*, *C*, *c*_0_ and *k* are arbitrary constants, *ω*(*θ*) is any integrable function, erfc(*y*) is error function and *c*_*n*_, *n* = 1, 2, ⋯ are constants given by the initial or boundary conditions of Eq (35). *τ* = ±1/2 is given in Eq (31). It is noticed that the last solution in Eq (34) yields an exact solution to the Boussinesq- Burgers equation with containing an arbitrary function, which may give more freedom to solve related problems of the equations [7].

### Some examples of new exact solutions

#### General form exact solutions

In Case II, substituting any solution of heat Eq (35) into Eq (12) and assembling them in Eq (14) with *i* = 2, we obtain exact solutions to Eqs (8) and (9). As examples, we present four exact solutions (*u*_*j*_, *v*_*j*_)(*j* = 1,2,3,4) of Eqs (8) and (9) corresponding to the first four solutions in Eq (34). Here we have

1. $\xi (t,x)=A\exp(-{\left(x+at\right)}^{2}/4\tau t)/\sqrt{t}+B,(t,x)\in R^{2},\tau =\pm 1/2$:
  $$\begin{array}{ccc} u_{1}\left(t,x\right) & = & \frac{1}{4[Ath_{1}\left(t,x\right)+Bt^{3/2}]}\left[at\left(-2B\sqrt{t}+A\left(\delta -2\right)h_{1}\left(t,x\right)\right)+A\delta xh_{1}\left(t,x\right)\right]\\ & & +\frac{A(at+x)}{2t^{3/2}}h_{1}\left(t,x\right)\phi_{2}\left(\xi \right),\\ v_{1}\left(t,x\right) & = & \frac{Ah_{1}\left(t,x\right)}{4{\left[Ath_{1}\left(t,x\right)+Bt^{3/2}\right]}^{2}}[-B\delta \sqrt{t}\left(\pm t+a^{2}t^{2}+2atx+x^{2}\right)\\ & & +Ah_{1}\left(t,x\right)\left(\mp \delta t+2\nu a^{2}t^{2}+4a\nu tx+2\nu x^{2}\right)]\\ & & \pm \frac{Ah_{1}\left(t,x\right)}{2\left[Ah_{1}\left(t,x\right)+B\sqrt{t}\right]t^{5/2}}[\mp B\sqrt{t}\left(\pm t+a^{2}t^{2}+2atx+x^{2}\right)\pm Ah_{1}\left(t,x\right)(t\left(2a\left(\delta -1\right)x\mp 1\right)\\ & & +\left(\delta -1\right)\left(a^{2}t^{2}+x^{2}\right)]\phi_{2}\left(\xi \right)+\frac{A^{2}{\left(at+x\right)}^{2}2}{2t^{3}}h_{1}^{2}\left(t,x\right)\phi_{2}^{2}\left(\xi \right), \end{array}$$
  where *A*, *B* are arbitrary constants, and *h*_1_(*t*, *x*) = exp[−(*x*+*at*)^2^/4*τt*].
2. *ξ*(*t*, *x*) = *A*exp(−*τk* ^2^ *t*)cos(*k*(*x*+*at*)+*B*)+*C*, (*t*, *x*) ∈ *R* ^2^, *τ* = ±1/2:
  $$\begin{array}{ccc} u_{2}\left(t,x\right) & = & -\frac{1}{4\xi (t,x)}\left[2aC+2aA\cos\left(\xi_{0}\right)h_{2}\left(t,x\right)\pm Ak\delta \sin\left(\xi_{0}\right)h_{2}\left(t,x\right)\right]\mp \frac{1}{2}Ak\sin\left(\xi_{0}\right)h_{2}\left(t,x\right)\phi_{2}\left(\xi \right),\\ v_{2}\left(t,x\right) & = & \frac{1}{4\xi^{2}\left(t,x\right)}[Ak^{2}h_{2}\left(t,x\right)(C\delta \cos\left(\xi_{0}\right)+A\left(\delta +\nu -\nu \cos\left(2\xi_{0}\right)h_{2}\left(t,x\right)\right]\\ & & +\frac{1}{2}Ak^{2}h_{2}\left(t,x\right)\left[\cos\left(\xi_{0}\right)+\frac{A\delta \sin^{2}\left(\xi_{0}\right)h_{2}\left(t,x\right)}{\xi (t,x)}\right]\phi_{2}\left(\xi \right)+\frac{1}{2}Ak^{2}h_{2}^{2}\left(t,x\right)\sin^{2}\left(\xi_{0}\right)\phi_{2}^{2}\left(\xi \right), \end{array}$$
  where *A*, *B*, *C* are arbitrary constants, and *ξ*_0_ = *B*+*k*(*x*+*at*), *h*_2_(*t*, *x*) = exp(−*τk* ^2^ *t*).
3. *ξ*(*t*, *x*) = *A*exp(−*k*(*x*+*at*))cos(*kx*+*k*(*a*−2*τk*)*t*+*B*)+*C*, (*t*, *x*) ∈ *R* ^2^, *τ* = ±1/2:
  $$\begin{array}{ccc} u_{3}\left(t,x\right) & = & \frac{-2a\left[C+A\cos\left(\xi_{0}\right)h_{3}\left(t,x\right)\right]\mp Ak\delta h_{3}\left(t,x\right)h_{4}\left(t,x\right)}{4\xi (t,x)}\mp \frac{1}{2}Akh_{3}\left(t,x\right)h_{4}\left(t,x\right)\phi_{2}\left(\xi \right),\\ v_{3}\left(t,x\right) & = & \frac{Ak^{2}h_{3}\left(t,x\right)}{4\xi^{2}\left(t,x\right)}\left[-2C\delta \sin\left(\xi_{0}\right)+A\left(\delta +2\nu +2\nu \sin\left(2\xi_{0}\right)\right)h_{3}\left(t,x\right)\right]\\ & & +\;\frac{Ak^{2}h_{3}\left(t,x\right)}{2\xi (t,x)}\left[-2C\sin\left(\xi_{0}\right)+A\left(\delta +\left(\delta -1\right)\sin\left(2\xi_{0}\right)\right)h_{3}\left(t,x\right)\right]\phi_{2}\left(\xi \right)+\frac{1}{2}A^{2}k^{2}h_{3}^{2}\left(t,x\right)h_{4}^{2}\left(t,x\right)\phi_{2}^{2}\left(\xi \right), \end{array}$$
  where *A*, *B*, *C* are arbitrary constants, and *ξ*_0_ = *B*+*kx*+*kt*(*a*−2*kτ*), *h*_3_(*t*, *x*) = exp[−*k*(*x*+*at*)], *h*_4_(*t*, *x*) = cos*ξ*_0_+sin*ξ*_0_.
4. $\xi (t,x)=c_{0}\text{erfc}(\frac{x+at}{2\sqrt{\tau t}}),t>0,x+at>0,\tau =1/2:$
  $$\begin{array}{ccc} u_{4}\left(t,x\right) & = & -\frac{a}{2}+\frac{\delta h_{1}\left(t,x\right)}{2\sqrt{2\pi t}\mathrm{erfc}\left(\xi_{0}\right)}+\frac{c_{0}}{\sqrt{2\pi t}}h_{1}\left(t,x\right)\phi_{2}\left(\xi \right),\\ v_{4}\left(t,x\right) & = & \frac{h_{1}^{2}\left(t,x\right)}{4\pi t^{3/2}{\mathrm{erfc}}^{2}\left(\xi_{0}\right)}\left[2\sqrt{t}\left(\delta +2\nu \right)-\sqrt{2\pi }\delta h_{1}^{-1}\left(t,x\right)\left(x+at\right)\mathrm{erfc}\left(\xi_{0}\right)\right]\\ & & +\;\left[\frac{c_{0}\delta h_{1}^{2}\left(t,x\right)}{\pi t\mathrm{erfc}(\xi_{0})}-\frac{c_{0}\sqrt{2}\left(x+at\right)h_{1}\left(t,x\right)}{2\sqrt{\pi }t^{3/2}}\right]\phi_{2}\left(\xi \right)+\frac{c_{0}^{2}h_{1}^{2}\left(t,x\right)}{\pi t}\phi_{2}^{2}\left(\xi \right), \end{array}$$
  where *c*_0_ is an arbitrary constant, and $\xi_{0}=\frac{x+at}{2\sqrt{\tau t}}.$

In all of above cases, *ϕ*_2_(*ξ*) is given in Eq (5).

By the same manner, from the rest two solutions in Eq (34), we should obtain additional exact solutions in different styles to the Boussinesq-Burgers. Due to the lack of space, we omit the reasoning and the solution expressions.

**Remark 6**: In author’s knowledge, this is the first time showing these kinds of exact solutions to Eqs (8) and (9). These solutions can not be obtained by other simplest equation methods [18–25].

#### The multi-soliton solutions

Using the connection between solutions of Eqs (8), (9) and (35), we can get the multi-soliton solutions of Eqs (8) and (9). It is easy to check that Eq (35) admits solutions

$$\begin{array}{c} \xi \left(t,x\right)=A_{0}+\sum_{i=1}^{n}A_{i}\exp\left[k_{i}x+\left(\tau k_{i}^{2}+ak_{i}\right)t+B_{i}\right],\;\;\tau =\pm 1/2, \end{array}$$

for arbitrary constants *k*_*i*_, *A*_*i*_, *B*_*i*_(*i* = 0, 1, ⋯, *n*) and positive integer *n*, which yield multi-soliton solutions of Eqs (8) and (9) given by Eqs (12) and (14) with *i* = 2.

#### The rational solutions

Suppose that Eq (35) has a solution in the form

$$\begin{array}{c} \xi \left(t,x\right)=\sum_{i=0}^{n}k_{i}\left(X\right)t^{i},X=x+at, \end{array}$$ (35)

for arbitrary positive integer *n*. Substituting Eq (35) into Eq (35) and setting the coefficients of *t* ^*i*^(*i* = 0, 1, ⋯, *n*) to be zero, we obtain the ODEs for *k*_*i*_(*X*) as $k_{n}^{\prime \prime }=0$ and $\alpha k_{j-1}^{\prime \prime }=k_{j},j=n,\cdots ,1$, which yield the solutions

$$\begin{array}{c} k_{i}\left(X\right)=\sum_{j=1}^{2(n+1-i)}\alpha_{j}\frac{X^{2(n+1-i)-j}}{[2(n+1-i)-j]!},i=0,1,2,\cdots ,n, \end{array}$$

for arbitrary constants *α*_*j*_. Substituting the solutions into Eq (35), we obtain the polynomial solutions of Eq (35), which yield rational solutions of Eqs (8) and (9) given by Eq (12) and (14) with *i* = 2.

## 4 Conclusions

In this paper, we proposed a new auxiliary equation method to look for exact solutions of NLEEs. There are two features of the discussed method which are different from other simplest equation methods [18]–[25]. Firstly, a solution expression with a variable coefficient is chosen and a special simplest nonlinear differential equations (a special ODE with variable coefficients) is taken as auxiliary equation. Secondly, the method can yield a Bäcklund transformation between the given NLEEs and the related constraint equation. By dealing with the constraint equation, we can derive infinite number of exact solutions for NLEEs. These solutions include the traveling wave solutions, non-traveling wave solutions, multi-soliton solutions, rational solutions and other types of solutions for NLEEs. According to the auxiliary equation and above two features of our method, we call the method as the generalized simplest equation method.

As application of our method, a great number of new exact solutions of the Boussinesq-Burgers equation are obtained. Essentially, the constraint equation is related to linear heat equation which result in new exact solutions of the Boussinesq-Burgers equation. These solutions are different from those solutins given in literatures [26–32]. It shows that our method is more flexible in finding more general form exact solutions and the method can be used for many other NLEEs in mathematical physics. As a result, our method effectively enhances the existing auxiliary equation methods, such as those given in [10, 16], which demonstrates that the proposed method is effective and prospective.

We have also tested other several commonly used auxiliary equations, such as Riccati equation [10, 11], the auxiliary equation of F-expansion method [12], to get exact solutions of the Boussinesq-Burgers equation in the frame of our method. Unfortunately, we can not get the results presented in the paper. What’s more, the relationship between the Boussinesq-Burgers equation and the linear heat equation can not be found. Hence the auxiliary Eq (3) has its own advantages which are not admitted by other auxiliary equations.

In addition, the true idea of our method makes one further consider finding more general exact solutions of other NLEEs by developing the existing methods, such as Tanh-method, the simplest equation method, etc, or designing new method, in which a connection between the constraint equation and other easily solved equation may be set up. It is an excellent topic for further research.
